# Nutrient Management Effects on Wine Grape Tissue Nutrient Content

**DOI:** 10.3390/plants11020158

**Published:** 2022-01-07

**Authors:** John L. Havlin, Robert Austin, David Hardy, Adam Howard, Josh L. Heitman

**Affiliations:** 1 Department of Crop & Soil Science, North Carolina State University, Raleigh, NC 27695, USA; reaustin@ncsu.edu (R.A.); amhoward@ncsu.edu (A.H.); jlheitman@ncsu.edu (J.L.H.); 2 North Carolina Department of Agriculture & Consumer Services, Agronomic Services Division, Raleigh, NC 27607, USA; david.hardy@ncagr.gov

**Keywords:** wine grapes, nutrient management, remote sensing, soil testing

## Abstract

With limited research supporting local nutrient management decisions in North Carolina grape (*Vitis vinifera*) production, field studies (2015–17) were conducted to evaluate late season foliar nitrogen (N) application on leaf and petiole N concentration and yeast assimilable N (YAN) in the fruit. Foliar urea (1% *v/v*) was applied at different rates and application times beginning pre-and post-veraison. Compared to soil applied N, late season foliar N substantially enhanced petiole N and grape YAN. Smaller split N applications were generally more effective in increasing YAN than single larger N rates. These data demonstrate the value of assessing plant N content at full bloom with petiole N analysis or remote sensing to guide foliar N management decisions. Additional field studies (2008–11) were conducted to evaluate pre-bud soil applied phosphorus (P) and potassium (K) effects on petiole P and K nutrient status. Fertilizer P and K were initially broadcast applied (0–896 kg P_2_O_5_ ha^−1^; 0–672 kg K_2_O ha^−1^) prior to bud-break in 2008–09 and petiole P and K at full bloom soil test P and K were monitored for three to four years after application. Soil test and petiole P and K were significantly increased with increasing P and K rates, which subsequently declined to near unfertilized levels over the sampling time depending on site and P and K rate applied. These data demonstrate the value of annually monitoring petiole P and K levels to accurately assess plant P and K status to better inform nutrient management decisions.

## 1. Introduction

Wine grape production is a rapidly growing industry in North Carolina (NC), which ranks 10th in the United States (US) in wine grape production, 7th in wine production, and represents a $2 billion total economic impact [1,2]. The dominant wine grapes produced in the western Piedmont and mountain regions of NC are *V. vinifera*, which includes the European varieties and both French and American hybrids [3]. As the NC wine industry expanded, the demand increased for research-based information to support management decisions. However, soil fertility and plant nutrition research supporting soil and plant diagnostic criteria were limited. Despite these limitations and driven by increasing demand for information, guidelines have been established based on research from other regions [4,5], which may not be appropriate for NC conditions.

Although soil testing traditionally is used to assess the potential for nutrient stress in annual crops, with perennial fruit crops, tissue analysis is considered a more reliable diagnostic tool to assess nutritional status [6]. Soil tests are essential to determine soil nutrient status prior to vineyard establishment. However, plant analysis is critical to monitoring plant nutrient supply each growing season. Both forms of analysis should be used to maintain optimum nutrient availability [7]. Most grape production regions recommend use of petiole sampling at full bloom, although if nutrient stress symptoms appear later in the growing season, veraison sampling guidelines have also been established [8,9]. For nitrogen (N), there are sufficient data to suggest that leaf blade analysis may provide more stable results, especially during full bloom [10,11], although petiole analysis is dominantly used in many regions.

Ultisols are the dominant soil order in the southeastern US and are commonly characterized by low pH and base saturation (BS) throughout the soil profile [12]. Low BS is mostly due to soil formed from parent material high in silica and low in basic cations, but is also due to intense leaching. Due to low pH and BS, NC soils are commonly low in plant-available calcium (Ca), magnesium (Mg), and potassium (K). In addition, low phosphorus (P) availability is due to low P-containing parent materials, intense weathering, and low solubility of Al-P minerals [13]. While adequate availability of all nutrients is essential to sustaining healthy vines and wine quality, the inherent properties of dominant soils in the V. vinifera region of NC suggest that aluminum (Al) toxicity and P, K, Ca, and Mg deficiency are important parameters to monitor.

Adequate N availability is required to support optimum grape yield and fruit quality [14]. Application of N fertilizers to vineyard soils can be an effective management tool, particularly where soil N status is low [3]. In N deficient soils, soil applied N may optimize yield and plant N content; however, in humid regions, growers are generally hesitant to soil apply N due to increased risk of excessive vine vigor. Under elevated N supply, canopy density (leaf area) increases, reducing airflow through the canopy and extending the duration of leaf and cluster wetness [14]. This change in microclimate increases the potential for Botrytis and other leaf and cluster diseases [4,5,15]. In addition, extensive shoot growth from full bloom to post-veraison often requires additional thinning to create an acceptable canopy microclimate for fruit and wood maturation [16].

In the southern US where excessive plant available water encourages vine growth and soils are low in organic matter [17], only low N rates are applied early in the growing season to avoid the potential negative consequences of excessive N fertilization [18,19]. Consequently, yeast assimilable N (YAN) in grape musts is frequently below the minimum threshold (140–150 mg N L^−1^) required to avoid incomplete fermentation [20,21,22,23]. Grape must YAN composition represents NH_4_^+^ and amino acids (except proline) and influences the extent of fermentation and formation of flavor compounds in the wine [24]. Wines produced from low-YAN musts are also prone to develop a disorder called atypical aging that occurs after bottling.

The problem of low YAN is not exclusive to NC. For example, 13% of the grape musts in California, Oregon (OR), and Washington (WA) vineyards were <140 mg N L^−1^ YAN [25]; 50% of musts selected from WA, OR, and Idaho vineyards had YAN values <150 mg N L^−1^ [26]. Although mono-or diammonium phosphate are commonly added to supplement grape must N levels prior to fermentation, wine flavors are generally inferior to wines with sufficient grape must YAN levels prior to fermentation [20,27] Therefore, it is more desirable to enhance YAN levels through vineyard N management practices than adding N during fermentation [28].

One potential solution to avoid excessive vine vigor associated with N applications in *V. vinifera* grapes grown in NC and other southeastern states is to foliar apply N in late-season to increase YAN while causing minimal changes to vine growth and disease potential. At veraison, a large part of N uptake is translocated to the grape clusters [29] Late-season soil applied N may not be effective in enhancing cluster N due to low surface soil moisture content [30], reducing N absorption by roots. If N uptake were increased with soil applied N at veraison, enhanced vine vigor is not desirable due to increased disease potential. In contrast, grape leaves are able to absorb N as urea and is usually taken up rapidly by the leaf cuticle [31].

In one of the early studies of late-season foliar N on wine quality, Lacroux et al. [32] demonstrated soil applied N increased vigor and Botrytis incidence, whereas, foliar N improved vine N status and enhanced aroma characteristics of Sauvignon blanc without increasing vigor or Botrytis susceptibility. Other recent studies confirm the positive effects of foliar N on increased YAN and wine aromatics [33,34,35]. In particular, foliar N was more effective in increasing juice YAN compared with early season soil applied N [36] These results confirm significant improvements in the aromatic profile and intensity of wines made from Tempranillo grapes treated with foliar N. Lower aromatic intensity and pronounced herbaceous flavors were observed in wines not treated with foliar N. In addition, foliar N increased grape amino acid concentrations, which improved must N composition and enhanced fermentation kinetics. The above studies demonstrate that the use of 1–2% (*v/v*) urea foliar applied at veraison shows considerable promise compared to traditional soil applied N.

In soil and plant nutrient surveys of numerous NC vineyards Havlin et al. [17] reported over nearly 70% of full-bloom petiole samples were below critical N levels, while approximately 20% and 30% were below critical P and K levels, respectively. The low N status of *V. vinifera* grown in NC is related to the minimal or no N applied by growers, a common practice used to reduce vine vigor and associated disease pressure. It is common to observe N deficiency symptoms on *V. vinifera* grapevines, especially from pre-veraison through harvest growth stages.

While only 30% or less of NC vineyards are P and/or K deficient, P and K fertilizer management decisions should be based on local research supporting interpretation of soil and plant analyses data [7]. When soil test and/or plant analysis levels are above established critical levels, no additional P or K fertilizers are needed. Unfortunately, few data are available to assess the relative plant tissue P/K response to P/K applications. With P, Janat et al. [37] reported two-year average petiole P increased from 0.21% to 0.30% with 100 kg P_2_O_5_ ha^−1^ applied annually. Using two *V. vinifera* varieties grown on P deficient soils, initial soil application of 300 kg P_2_O_5_ ha^−1^ increased full bloom petiole P from 0.8% to 1.2% in the first year, increasing to 1.8% in the second year, and decreasing to 1.5% in the third year after P application [38]. In contrast, higher K rates are generally used compared to P, although excessive K applications can reduce wine quality by increasing must pH or decreasing titratable acidity [39,40]. For example, concern for excessive K effects on wine quality in Virginia resulted in a reduction in soil test critical K levels and subsequent fertilizer K recommendations [41]. Using a French hybrid (cv. Foch) grown medium soil test K, sandy soil, full bloom petiole K increased from 2.8% to 3.2% K (average over five years) with annual applications of 600 kg K_2_O ha^−1^ [42]. In a survey of 60 vineyard growers in central India, Naraboli et al. [43] reported 2.50–2.75% petiole K at full bloom was associated with optimum yield and was achieved with an average 1000 kg K_2_O ha^−1^. After three years of annual applications of up to 200 kg K_2_O ha^−1^ to cv. Cabernet sauvignon in Brazil, Mehlich 1 K increased nearly 10-fold (50–464 mg kg^−1^ and full bloom petiole K from 2.0% to 3.6% [44].

Due to limited research resources available to establish critical N, P, and K plant tissue levels under NC conditions, critical nutrient levels (NC) were adopted from those established in other regions [4,5]. Despite this limitation, research information can be provided to assess relative plant nutrient response to N, P, and K application. Therefore, using established plant nutrient critical levels, vineyard managers can decide if additional nutrients are needed and should expect applied nutrients to increase plant nutrient levels to or above the critical levels. Therefore, the objectives of these studies were to: (1) evaluate late season foliar N application on YAN in the fruit, and (2) quantify soil and plant nutrient response to soil applied P and K.

## 2. Results and Discussion

### 2.1. Foliar N Studies: Plant N and Normalized Difference Vegetative Index (NDVI)

Soil properties (check plots) were typical of vineyard soils (relatively high P, K, micronutrients) in the Yadkin Valley Appellation (Surry Co., NC) with a previous history of manure applications (old dairy farm; Table 1). Soil pH (0–20 cm) was optimum (Site A) for *V. vinifera* grape production; however, at Site B soil pH was below optimum. At Site A, soil samples were also collected from the soil applied N plots, although results were not significantly different from soil test data obtained from the untreated plots, thus residual soil applied N was not detected in at either site (data not shown). Monthly rainfall and temperature data were relatively normal, although 2017 rainfall was 30% higher than the 50-year average (Table 2).

Leaf and petiole N analyses at full-bloom (prior to foliar N application) were necessary to establish plant N levels that could be used to assess foliar N need. At all sites in each year petiole and leaf N contents at full bloom were below established critical levels of 1.2–1.6% petiole N and 2.5–3.5% leaf N [3,45], although in each year leaf N was at or slightly above 3.5% N in site A (Table 3). No significant differences in plant N or NDVI were detected between treatment areas since foliar N applications did not begin until pre-veraison. Despite 100 kg N ha^−1^ soil applied at bud break, plant N was not affected in 2015. In 2016 with 200 kg ha^-1^ soil applied N, leaf N at both sites and petiole N at site A were slightly but significantly increased, although petiole N (site B) was not affected (Table 3). In 2017, soil applied N at both sites slightly but significantly increased petiole N, but no difference was observed in leaf N (Table 3). Compared to 2015, where a lower N rate was soil applied, the plant N data illustrate that soil N applied at relatively high rates may only slightly increase plant N content at full bloom. 

**Figure 1:**
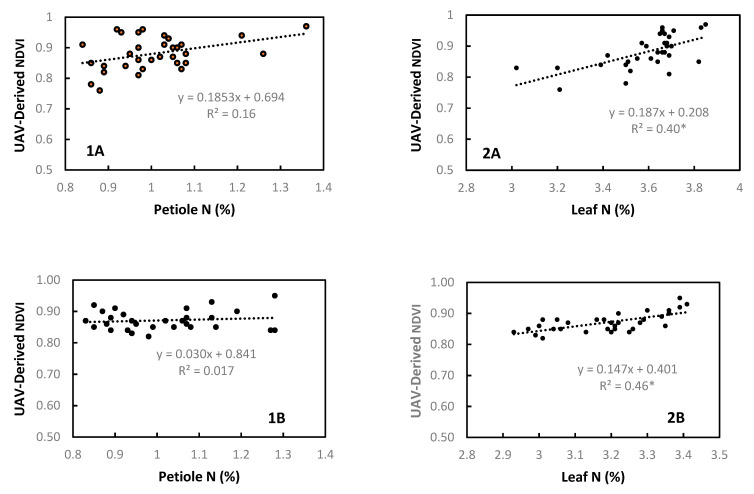
Relationship between petiole (1) and leaf (2) N and UAV-based NDVI at site A and B in 2016. * significant at *p* = 0.05. Similar results found in 2015 (site A, data not shown).

In order to develop foliar N recommendations based on UAV-acquired imagery, NDVI measurements should relate to leaf or petiole N content. There was no clear correlation between petiole N and NDVI in both 2015 (site A, data not shown) and 2016 (site A and B), which was expected since reflectance occurs dominantly on the leaf surfaces. In contrast, leaf N and NDVI were correlated, indicating a potential use for multispectral remote sensing to asses plant N status (Figure 1). However, due to the planophile leaf structure of most *V. vinifera* and the tendency for NDVI and other similar vegetative indexes to saturate as biomass increases, the ability to resolve differences at higher N concentrations and later in the season decreases [46]. This highlights the importance of the timing of flights and variability of leaf N. Comparatively, when only small differences in tissue N are present, UAV-based measurements are of less value and leaf or petiole sampling is required.

A contributing factor related to the difficulty in remotely measuring N content in 2017 was the limited grape leaf area within the canopy and an early morning flight time. After classifying the pixels into classes representing the grape canopy, soil, and surrounding grass, only 8% of the area was identified as grape canopy, the remaining 92% was bare soil or grass. Consequently, variations in leaf angle and leaf orientation had a greater effect on the resultant NDVI value compared to data collected in earlier years.

### 2.2. Foliar N Studies: Wine Grape Quality

In 2015, soil applied N (pre-bud break) had little effect on wine grape quality parameters compared to no N applied (Table 4). In contrast, foliar N significantly increased YAN and malic acid in grapes at harvest. A small increase in juice pH was also observed. These parameters generally increased with increasing N rate (0–44.8 × 1). However, the two treatments that resulted in the highest YAN levels were the 22.4 and 11.2 × 4 rates. The 44.8 and 22.4 × 2 treatments resulted in severe and moderate leaf edge burn, respectively, thus both were discontinued in 2016–17.

In contrast to 2015, the higher soil applied N rate in 2016–17 increased malic acid and YAN compared to the 0 N treatment (2016-site A and B; 2017-site A), whereas soil N did not significantly increase YAN in 2017 (site B) (Table 5). Jreij [47] reported maximum YAN with both soil and foliar applied N. No significant N treatment effects on Brix, pH, and TA were observed at either stie in 2016–17 (data not shown).

At both sites, increasing foliar N rate significantly increased YAN and malic acid compared to the “0” treatment, and were also significantly greater than with soil applied N (Table 5). Compared to the 0 N treatment, the 11.2×4 split N treatment increased 2016 YAN 58% and 49% at site A and B, respectively, while increasing YAN at both sites by nearly two-fold over the soil applied N treatment, respectively, in 2017. Similar differences between foliar and soil applied N on YAN were recently reported for *V. vinifera* cv. petit manseng [48]. The split N treatments generally exhibited a larger response in YAN and malic acid than single N application rates. For example, in 2016 the 11.2 × 2 treatment increased YAN by 24% and 12% over the single 22.4 rate at site A and B, respectively. In contrast, foliar N with these same treatments increased YAN by 15% and 17% at site A and B, respectively, in 2017. In both years, similar trends in malic acid were observed with these same N treatments, although the effects were greater at site A than site B. Using 15N, Lasa et al. [34] demonstrated that split N applications on Merlot and Sauvignon Blanc, especially post-veraison, were more effective in increasing N in grapes berries compared to single applications of low foliar N rates and soil applied N. Foliar N treatment effects on malic acid were similar to those on YAN, although the increases were not as pronounced (Table 5). Similar studies in British Columbia using three split applications of 12 kg N ha^−1^ each over three years on seven vineyards and five varieties consistently increased (35–245%) YAN [23]. Bavaresco et al. [49] also documented split applications of foliar urea during the growing season increased YAN compared to a single N application. The results of our foliar N experiments concur with many other recent studies documenting foliar N was effective in increasing diverse flavonoid contents in grapes and wines from N deficient vineyards [50,51,52,53].

### 2.3. P and K Studies

The vineyards included in the P and K nutrient response studies were selected for potential wine grape response to soil applied P and K (Table 6). The P sites were at or below the 28–32 mg kg^−1^ critical soil test P level, while the K sites were at or below the 120–130 mg kg^−1^ soil test K critical level [54]. Monthly growing season rainfall and temperature data were relatively normal, although at the Surry Co. site (1P09/1K09) rainfall was ~25% higher than the 50–year average in 2009 and 2011 (Table 7). In each year, growing season rainfall and temperature were relatively normal at 2P09 (Moore Co. site), while at 2K08, growing season weather conditions were similar to 50-year averages except in 2010 and 2011 where rainfall was 18% and 34% higher, respectively.

**Figure 2:**
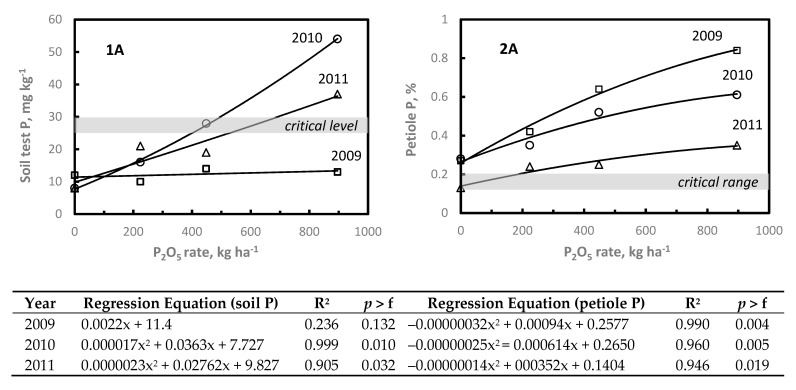

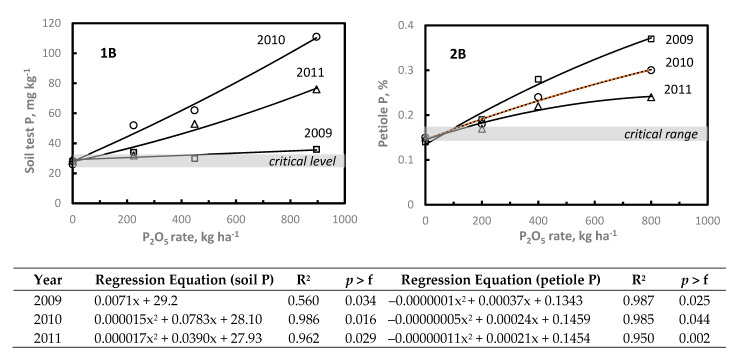
Influence of soil applied P_2_O_5_ on soil test P (1) and petiole P (2) content at 1P09 (A) and 2P09 (B) sites. Critical soil test level and critical nutrient range are displayed (shaded bar). Symbols for each year represent treatment means.

At both P sites (1P09 and 2P09), soil test P increased substantially in 2010 following P application in 2009 (Figure 2). With 896 kg P_2_O_5_ ha^−1^ applied in 2009, soil test P increased approximately four- and five-fold above initial soil test P levels at the 2P09 and 1P09 sites, respectively. At 1P09, 448 and 896 kg P_2_O_5_ ha^−1^ were needed to reach or exceed the critical soil test P level in 2010; whereas by 2011 soil test P exceeded the critical P level with only the highest P_2_O_5_ rate. Since the initial soil test P at 2P09 was at the critical level, soil test P exceeded 28–32 mg P kg^−1^ with all P rates in 2010, and subsequently declined by nearly 50% by 2011 (Figure 2). Although the soil test P response to applied P_2_O_5_ was curvilinear (Figure 2), approximately 17 kg and 9.5 kg P_2_O_5_ ha^−1^ were required to increase soil test P 1 mg kg^−1^ at 1P09 and 2P09 sites, respectively. These data were similar to those reported for similar Ultisol soils in NC [55,56].

Although initial soil test P was below critical P level at 1P09, petiole P concentration was at the critical petiole P range with no P applied and significantly increased with increasing P rate (Figure 2). In 2009 at 1P09, petiole P concentration increased two-, three-, and four-fold above the petiole P concentration with no P applied. In contrast at 2P09, petiole P concentration in 2009 was below the critical P range with the 0 and 224 kg P_2_O_5_ ha^−1^ treatments, although initial soil test P was at the critical level. Using similar P rates, Janat et al. [37] and Conradie et al. [57] reported similar increases in petiole P with soil applied P. With 448 kg and 896 kg P_2_O_5_ ha^−1^ applied, petiole P increased nearly 1.5- and 2-fold above the critical petiole P range in 2009, decreasing by nearly 50% in 2010, and by 2011 petiole P had declined to the critical P range. Thus, even at 896 kg P_2_O_5_ ha^−1^ petiole P was just slightly above critical petiole P three years after P application.

**Figure 3:**
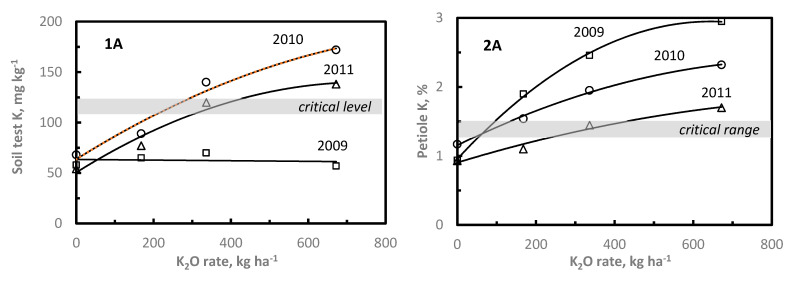

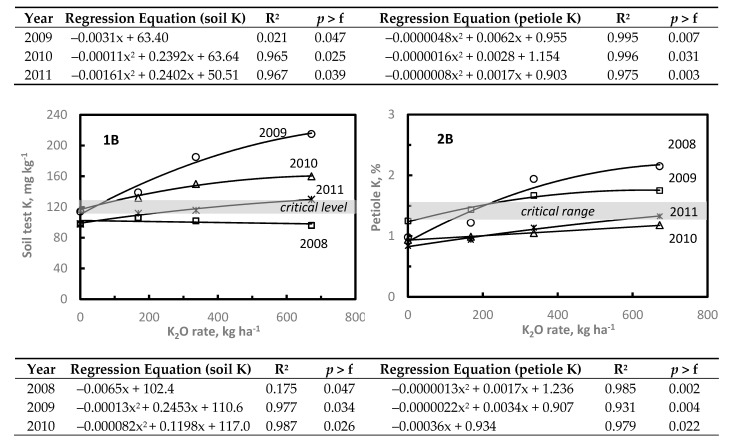
Influence of soil applied K_2_O on soil test K (1) and petiole K (2) content at 1K09 (A) and 2K08 (B) sites. Critical soil test level and critical nutrient range are displayed (shaded bar). Symbols for each year represent treatment means.

At both K sites, soil test K significantly increased with increasing K rate (Figure 3). With the 672 kg K_2_O ha^−1^ treatment, soil test K increased approximately two- and three-fold above the 0 K treatment at 2K08 and 1K09 sites, respectively. Similar to the P sites, soil test K response to applied K_2_O was curvilinear (Figure 3), where an average of 5.6 kg and 6.5 kg K_2_O ha^−1^ were required to increase soil test K 1 mg kg^-1^ at 1K09 and 2K08 sites, respectively. As with P, the K rate needed to increase soil test K by 1 mg kg^−1^ is highly variable depending type and quantity of soil clay minerals. A recent summary reported a range of 4–20 kg K_2_O ha^−1^ to increase soil test K by 1 mg kg^−1^ [58]. At 1K09, 336 kg K_2_O ha^−1^ were needed to reach or exceed the critical soil test K level; whereas only 168 kg K_2_O ha^−1^ were needed at the 2K08 site. Two years after K application at 1K09, soil test K had declined to or below the critical K level with all treatments except for the highest K rate which remained slightly above the critical K level. While soil test K declined following K application at the 2K08 site, soil test K remained above the critical K level two years after K application. However, in the third year, soil test K was at or below the critical level with all K treatments.

Increasing K rate significantly increased petiole K above the critical K range at both sites in 2009, although the response in petiole P was greater at 1K09 compared to 2K08. For example at the 672 kg K_2_O ha^−1^ rate, petiole K increased two- and three-fold above petiole K concentration with no K applied at 1K09 and 2K08, respectively (Figure 3). This difference may be due to higher initial soil test K level at 2K08. Similar responses were reported by Neilsen et al. [42] and Ciotta et al. [44]. In subsequent years, petiole K at 1K09 decreased by approximately 50% in 2010, while in 2011 petiole K had declined to the critical range with 168 kg K_2_O ha^−1^ treatment and remained slightly above the critical K range at the higher K rates. Similarly at site 2K08, petiole K increased to or above the critical K range in 2009, and although still above the critical range, petiole K declined by approximately 50% by 2010. By 2011, petiole K had declined to the critical level or slightly below with all treatments. 

## 3. Materials and Methods

### 3.1. Foliar N Studies

Based on previous nutrient survey studies in NC vineyards, a vineyard was selected that exhibited low petiole and leaf N concentrations at full bloom and veraison [17]. The experimental sites were located at Shelton Vineyards in Surry Co., NC. In 2015, the site was on a Fairview sandy clay loam (fine, kaolinitic, mesic Typic Kanhapludults), and in 2016–17, an additional site was added on a similar soil type (five site-years). Both sites were planted in 2006 with Merlot cv. on 101–14 rootstock. Vineyard rows were 3 m apart with 1.5 m vine spacing within rows. Ground cover between rows was tall fescue (*Festuca arundinacea*) with 1 m bare surface maintained under the vine canopy. At each site, treatments were applied to 3 m × 15 m plots, representing 10–12 vines per plot. The experimental design was a randomized complete block with four replications. Monthly growing season (March–October) rainfall and temperature data were obtained from nearest weather station in Mt. Airy, NC (US Climate Data 2021, version 3.0).

Treatments (2015) included single applications of 11.2, 22.4 kg, and 44.8 kg N ha^−1^ 22.4 kg and 44.8 kg N ha^−1^ applied at 11.2 kg and 22.4 kg N ha^−1^ (2 splits), respectively; and 44.8 kg N ha^−1^ split applied at 11.2 kg (4 splits) and 22.4 (2 splits) kg N ha^−1^, respectively (Table 8). For the soil N treatment, 100 kg N ha^−1^ as urea was broadcast applied in early March, prior to budbreak. In subsequent years (2016–17) soil applied N was increased to 200 kg N ha^−1^, while foliar N treatments deviated slightly due to salt damage to foliage observed in 2015 with the 44.8 kg N ha^−1^ rate applied once or in two split applications.

In early February, soil samples were collected prior to bud-break (early-mid March) from each site at 0–10 and 10–20 cm depths. Four cores were randomly collected from each untreated plot and composited, from which a subsample was air dried and sent to the NC Department of Agriculture and Consumer Services Laboratory for analysis [54].

Prior to application of the foliar N treatments, plant tissue (petiole and leaf) samples (40–50) were collected at full bloom (pre-treatment) from opposite the first or second cluster from the bottom of the shoot in each treatment area or plot. Petioles were immediately separated from lamina and both placed in separate labeled paper bags or envelopes. Tissue samples were dried in a forced air oven at 27 °C to 32 °C for 24 h, and submitted to the NC Department of Agriculture and Consumer Services Laboratory for total nutrient analysis (54).

The potential for remotely measuring plant N status using a Normalized Difference Vegetative Index (NDVI) was also evaluated. An Unmanned Aerial Vehicle (UAV) was used to capture multispectral imagery between 2015 and 2017 [59]. Flights were conducted using a DJI Phantom 3 drone (Da-Jiang Innovations, Shenzhen, China) and Parrot Sequoia (Parrot, Paris, France) four-band multispectral sensor. The Sequoia sensor measures radiance in the green: 530–570 nm, red: 640–680 nm, red-edge: 730–740 nm, and near-infrared: 770–810 nm wavelengths.

Images were collected using pre-programmed flights 50 m above ground level with 70% side-overlap and 80% forward overlap. Ground control points were placed in the vineyard before each flight and used during post-processing to enhance the geometric accuracy of the orthomosaics. Orthomosaics were developed using photogrammetric software (Agisoft Metashape, St. Petersburg, Russia).) using default processing parameters and the recommended processing workflow. Flights were conducted on 20 May 2015; 24 May 2016; and 11 May 2017, on the same day as plant tissues were collected for analysis. 

The georeferenced orthomosaics were used to calculate the fractional green-leaf area and an average NDVI for each plot. Plot boundaries were developed in a Geographic Information System (ESRI—ArcGIS 10.8, Redlands, CA, USA) [60] using the measured dimensions of each plot and the orthomosaic. A supervised maximum likelihood classifier [61] was used to segment and classify pixels into grass, bare soil, shadows, and grape-leaves. Pixels classified as grape-leaves were summed and used to calculate the fractional green-leaf area for each plot and used as a proxy to estimate canopy ‘vigor’ and biomass during analysis. NDVI was calculated using pixels classified as green-leaf area to assure NDVI values were representative of the vinifera leaf-canopy alone. NDVI is based on the measurement of red and near-infrared (IR) wavelengths described by:

NDVI = (Red − NearIR)/(Red + NearIR)(1)

These two wavelengths are known to correlate to both plant biomass and N content and are often related to plant ‘vigor’ [62,63,64]. Descriptive statistics for NDVI were calculated using the raster analysis and zonal statistics available in ArcGIS. NDVI values from 2016 and 2017 represent uncalibrated radiometric surface reflectance and consequently analyzed independently by year.

Foliar N treatments (Table 8) were applied with a backpack CO_2_ sprayer (R and D Sprayers, Inc., Opelousas, LA, USA) equipped with 4–80 °C flat spray nozzles on 0.30 m spacing. The 1.2 m spray boom was held vertically along each side of the treatment row to facilitate optimum canopy coverage. Urea solution (20% N) was diluted with variable amounts of distilled H_2_O to prepare 1650 mL of final N solution applied to each treatment. 

At harvest, ~8–10 clusters were collected from each plot and immediately placed in a cooler under dry ice. Samples were delivered immediately after harvest to the Appalachian State Chemistry and Fermentation Service Lab, where grape juice was analyzed for pH, total acidity (TA), Brix, malic acid, and YAN [65]. Juice YAN was determined by summing primary amino acid-N obtained by HPLC analysis and ammonia-N by an enzymatic assay [66]. Plant N and fruit quality data were analyzed using General Linear Models routine in SAS 9.4 (SAS Institute, Cary, NC) [67]. Treatment effect means for measured parameters were compared using Fisher’s protected LSD (*p* < 0.05). 

### 3.2. Soil P and K Studies

Field studies were initiated to evaluate the response in wine grape petiole P and K concentrations to soil applied P and K. Based on soil and plant nutrient survey data collected in 2006–08 [17], four vineyard sites were identified with potential vine response to applied P and K (Table 9). In 2008, one site was selected to represent the potential for low soil K at Hanover Park Vineyard (Yadkinville, Yadkin Co., NC, USA) and in 2009 three additional vineyards were identified (two P sites (Round Peak Vineyard, Dobson, Surry Co. NC, USA; Black Rock Vineyard, Sanford, Moore Co. NC, USA) and 1 K site (Round Peak Vineyard, Dobson, Surry Co., NC, USA). Four additional sites were used in the four-year P and K study. However, these data are not included since the initial soil test P and K levels exceeded established critical levels [44]. Monthly growing season (March-October) rainfall and temperature data were obtained from nearest weather stations in Mt. Airy, NC (1P09, 1K09), Sanford, NC (2P09), and Yadkinville, NC (2K08) [US Climate Data 2021, version 3.0].

At each site-year, 20 m rows were used per treatment with treatments arranged in a randomized complete block design with four replications. Plots were one vine row in a 3 m × 20 m plot, where ground cover between rows was tall fescue (*Festuca arundinacea*) with 1 m bare surface maintained under the vine canopy. In February of the 2008 and 2009 studies, soil samples (0–20 cm depth) were collected in each plot prior to treatment application, and again in the amended region each subsequent year after application to evaluate influence of treatments on soil test P and K. Soil samples were analyzed using standard soil analysis methods [54]. At the P sites, triple super phosphate (0–46–0) was applied at 0, 224, 448, and 896 kg P_2_O_5_ ha^−1^. At the K sites, potash (0–0–60) was applied at 0, 168, 336, and 672 kg K_2_O ha^−1^. After soil samples were collected, P and K treatments were broadcast applied about 40 cm on either side of the row without incorporation. 

Plant tissue (petiole and leaf) samples opposite the first or second flower cluster from the bottom of the shoot were collected at full bloom (20–28 May) (~40 petiole/leaf subsamples). Petioles were immediately separated from leaf blades and both placed in separate labeled paper bags or envelopes. Tissue samples were dried in a forced air oven at 80 to 90 °F for 24 h and submitted to the NC Department of Agriculture and Consumer Services Laboratory for total nutrient analysis [54]. Since NC vineyards utilize petiole analysis to assess vine nutrient status only the petiole P and K results are shown.

Analysis of variance for soil test and plant tissue data was performed using the General Linear Model procedure in SAS Version 9.1 (SAS, Cary, NC, USA) [67]. Mean comparisons were performed with Least Significance Difference (LSD) at a probability level of 0.05.

## 4. Conclusions

The purpose of these studies was to quantify the effect of foliar applied N on selected N parameters in wine grapes related to wine quality, and to evaluate petiole P and K response to soil applied fertilizer P and K.

Petiole N content was below the critical level (1.2–1.6%) at each site used in the foliar N studies, which suggest that foliar N applied pre- and post-veraison could significantly improve grape N content and other parameters critical to enhancing flavor compound concentrations in wine grapes. Increasing foliar N rates generally increased YAN, while split N applications generally increased wine grape quality parameters to a greater extent than single foliar applied N rates. Pre-bud break soil applied N had little or no effect on wine grape quality unless applied at elevated rates. These preliminary data also demonstrate the potential use of UAV-based remote sensing in assessing N status in the vineyard. Although UAV’s provide a unique opportunity to capture images at resolutions needed to detect plant N status, sensitivity of the measurements can be affected early in the season when there is little growth. As this analysis suggests, if this technology is to detect early-season plant N in V. vinifera, timing of flights should correspond with adequate canopy development to providing robust, reliable measurements. Therefore, identifying N deficient grape plants at full bloom by either plant sampling/analysis or through remote sensing can direct the vineyard manager to initiate late-season foliar N management to improve wine grape quality.

The P and K response studies were located on soils testing at or below critical soil test P or K levels. At each site, increasing P or K rate increased soil test and petiole P and K levels. In the two- or three-years after application, petiole P and K declined to at or below critical levels; however, at the highest P or K rates petiole P or K levels remained at or above the critical nutrient range. Since plants accumulate nutrients throughout the rooting depth, it is difficult to base P or K management decisions on soil test P or K. Therefore, these data illustrate the importance of annual petiole analysis to determine when petiole P or K levels decline to or below established critical levels warranting application of P or K. Although additional P and K response studies representing a wider range in clay content and other soil properties are needed to establish specific P and K rates required to increase petiole P or K above established critical levels. Based on these limited data, growers should regularly monitor petiole P/K, and if below the critical levels soil applied 200–300 kg P_2_O_5_ ha^−1^ and 300–400 kg K_2_O ha^−1^. Subsequent P or K rates should be adjusted depending in petiole P/K the year of or following application. 

## Figures and Tables

**Table 1:** Surface soil properties associated the two field sites used in the 2015-17 N studies.

Site	Depth	OM	CEC	BS	pH	Ca	Mg	P	K	S	Mn	Zn	Cu
	cm	%	cmol kg^−1^	%		%		mg kg^−1^					
2015													
A	0–10	0.48	9.0	82	5.9	61	18	74	123	17	23	22	12
	10–20	0.38	7.5	80	6.4	54	21	21	65	14	9	9	4
2016													
A	0–10	0.43	8.9	77	5.7	52	22	86	115	17	24	21	11
	10–20	0.36	6.9	84	6.2	56	21	18	67	15	13	7	3
B	0–10	0.60	5.0	65	5.4	43	18	76	119	15	17	10	9
	10–20	0.47	4.7	66	5.4	42	19	34	107	25	10	5	6
2017													
A	0–10	0.40	8.4	81	5.8	54	24	65	105	19	23	18	9
	10–20	0.33	6.5	87	6.4	57	28	11	57	19	11	6	2
B	0–10	0.36	4.8	70	5.5	44	20	79	173	19	19	10	11
	10–20	0.17	4.4	67	5.4	40	21	10	126	35	5	2	2

**Table 2:** Growing season rainfall and temperature at foliar N study sites in Surry Co. NC (2015–17).

Month	Precipitation				Temperature (max-min)			
	2015	2016	2017	50-yr Average	2015	2016	2017	50-yr Average
	mm				°C			
March	69	36	109	105	15–1	19–3	15–0	15–1
April	138	98	211	96	21–6	21–5	22–8	21–4
May	62	164	237	108	27–11	23–11	24–11	24–9
June	212	54	98	113	30–17	30–14	28–14	29–15
July	125	108	145	130	31–18	32–18	31–18	31–17
August	128	249	57	99	30–16	30–19	29–16	30–17
September	69	101	83	102	27–14	29–16	26–12	27–12
October	144	24	177	84	22–7	23–8	22–7	21–6
Total	948	833	1118	836				

**Table 3:** Foliar and soil applied N effects on plant N and NDVI at full bloom ^1^.

	2015 (Site A)			2016 (Site A)			2016 (Site B)			2017 (Site A)		2017 (Site B)	
N Treatment ^1^	Plant N			Plant N			Plant N			Plant N		Plant N	
	Petiole	Leaf	NDVI	Petiole	Leaf	NDVI	Petiole	Leaf	NDVI	Petiole	Leaf	Petiole	Leaf
kg ha^−1^	%			%			%					%	
0	0.97	3.53	0.84	0.98 ^a^	3.55 ^a^	0.86	1.00	3.19 ^a^	0.88	0.97 ^a^	3.75	0.92 ^a^	3.06
11.2	0.95	3.59	0.84	1.06 ^a^	3.66 ^a^	0.92	0.90	3.10 ^a^	0.87	1.03 ^a^	3.66	1.03 ^a^	3.11
22.4	0.93	3.59	0.83	0.94 ^a^	3.51 ^a^	0.85	0.93	3.25 ^ab^	0.89	1.08 ^a^	3.74	1.03 ^a^	3.08
11.2 × 2	0.97	3.63	0.86	0.95 ^a^	3.68 ^a^	0.87	1.05	3.24 ^ab^	0.87	1.03 ^a^	3.61	0.97 ^a^	3.07
44.8	0.92	3.65	0.83										
22.4 × 2	0.88	3.61	0.84										
11.2 × 3										1.05 ^a^	3.66	1.05 ^a^	3.14
11.2 × 4	0.93	3.74	0.86	1.03 ^a^	3.64 ^a^	0.90	1.05	3.03 ^a^	0.85	1.01 ^a^	3.54	0.96 ^a^	3.07
Soil N	0.96	3.57	0.85	1.16 ^b^	3.75 ^ab^	0.91	1.02	3.35 ^b^	0.89	1.19 ^b^	3.81	1.13 ^ab^	3.18
*p > f*	ns	ns	ns	0.018	0.025	ns	ns	0.043	ns	0.026	ns	0.038	ns

^1^ means followed by the same letter are not significant at *p* > 0.05.

**Table 4:** Foliar and soil applied N effects on selected wine grape quality parameters in 2015 (site A) ^1^.

N Treatment ^1^	Brix	pH	Titratable Acidity	Malic Acid	YAN
			g L^−1^	g L^−1^	mg L^−1^
0	21.2 ^a^	3.37 ^a^	4.14 ^a^	2.24 ^a^	143.7 ^a^
11.2	21.4 ^a^	3.41 ^a^	4.22 ^a^	2.41 ^b^	170.6 ^b^
22.4	21.4 ^a^	3.60 ^a^	4.36 ^a^	2.83 ^d^	217.5 ^c^
11.2 × 2	21.5 ^a^	3.52 ^a^	4.10 ^a^	2.60 ^c^	187.7 ^b^
44.8	21.1 ^a^	3.46 ^a^	4.25 ^a^	2.56 ^c^	194.5 ^bc^
22.4 × 2	21.7 ^a^	3.48 ^a^	4.19 ^a^	2.51 ^bc^	187.9 ^b^
11.2 × 3					
11.2 × 4	21.0 ^a^	3.63 ^a^	4.13 ^a^	2.91 ^d^	221.1 ^c^
Soil N	21.3 ^a^	3.44 ^a^	4.09 ^a^	2.30 ^a^	149.3 ^a^
*p > f*	ns	ns	ns	<0.0001	<0.0001

^1^ means followed by the same letter are not significant at *p* > 0.05.

**Table 5:** Foliar and soil applied N effects on wine grape quality parameters in 2016–2017 ^1^.

	2016				2017			
	Site A		Site B		Site A		Site B	
N Treatment ^1^	Malic Acid	YAN	Malic Acid	YAN	Malic Acid	YAN	Malic Acid	YAN
	g L^−1^	mg L^−1^	g L^−1^	mg L^−1^	g L^−1^	mg L^−1^	g L^−1^	mg L^−1^
0	2.28 ^a^	183 ^a^	2.19 ^a^	185 ^a^	2.22 ^a^	135 ^a^	2.24 ^a^	126 ^a^
11.2	2.48 ^a^	218 ^b^	2.42 ^b^	181 ^a^	2.38 ^a^	166 ^b^	2.46 ^b^	151 ^a^
22.4	2.40 ^a^	209 ^b^	2.24 ^a^	194 ^ab^	2.43 ^a^	187 ^c^	2.38 ^a^	191 ^b^
11.2 × 2	2.95 ^b^	260 ^d^	2.48 ^b^	218 ^b^	2.72 ^b^	216 ^d^	2.54 ^b^	223 ^b^
44.8								
22.4 × 2								
11.2 × 3					2.77 ^b^	237 ^d^	2.65 ^c^	245 ^c^
11.2 × 4	2.77 ^b^	290 ^e^	2.77 ^c^	275 ^c^	2.88 ^bc^	265 ^e^	2.81 ^c^	242 ^c^
Soil N	2.65 ^b^	233 ^c^	2.39 ^b^	201 ^b^	2.60 ^b^	193 ^c^	2.41 ^b^	138 ^a^
*p* > *f*	0.023	<0.001	0.021	<0.001	<0.001	<0.001	<0.001	<0.001

^1^ means followed by the same letter are not significant at *p* > 0.05.

**Table 6:** Soil properties of sites used in the P and K response studies (2008–2011).

Site Designation	Input	pH	CEC	BS	HM	P	K	Ca	Mg	Mn	Zn	Cu
			cmol kg^−1^	%		mg kg^−1^						
**P sites**												
1P09	P	6.4	6.7	83	0.40	11	159	719	227	8	8	1
2P09	P	5.3	6.3	58	0.82	22	145	477	181	76	1	2
**K sites**												
1K09	K	5.7	5.3	70	0.42	39	68	465	159	7	8	1
2K08	K	6.4	9.4	92	0.21	47	99	1109	369	18	5	1

**Table 7:** Growing season rainfall and temperature at P and K (2008–2011).

**1P09 and 1K09 Sites (Surry Co. NC)**										
**Month**	**Precipitation**					**Temperature (max-min)**				
	**2009**	**2010**		**2011**	**50-yr average**	**2009**	**2010**		**2011**	**50-yr average**
	mm					°C				
March	101	128		179	105	14–1	15–1		15–1	15–0
April	119	48		117	96	21–4	24–5		22–6	21–4
May	205	153		162	108	23–12	26–12		25–11	24–9
June	179	61		167	113	29–16	31–17		31–15	29–15
July	169	89		107	130	29–15	32–18		32–19	31–17
August	123	114		70	99	30–17	30–18		31–17	30–17
September	75	104		144	102	25–13	29–12		26–14	27–12
October	60	81		98	84	20–6	24–6		21–5	21–6
Total	1033	780		1044	836					
**2P09 Site (Moore Co. NC)**										
**Month**	**Precipitation**					**Temperature (max-min)**				
	**2009**	**2010**		**2011**	**50-yr average**	**2009**	**2010**		**2011**	**50-yr average**
	mm					°C				
March	146	79		85	101	19–4	18–2		20–4	18–3
April	31	29		52	73	25–8	27–7		26–9	24–8
May	85	85		109	89	27–14	28–16		28–14	27–13
June	43	73		72	118	31–18	33–20		33–18	31–18
July	178	168		166	131	32–19	34–21		34–21	32–20
August	117	109		123	120	31–15	32–17		32–17	31–19
September	101	131		62	105	28–10	31–8		28–7	28–9
October	43	18		64	91	23–5	25–2		22–4	23–4
Total	744	691		733	827					
**2K08 Site (Yadkin Co. NC)**										
**Month**	**Precipitation**					**Temperature (max-min)**				
	**2008**	**2009**	**2010**	**2011**	**50-yr average**	**2008**	**2009**	**2010**	**2011**	**50-yr average**
	mm					°C				
March	105	129	90	146	105	18–1	14–1	16–1	16–2	16–1
April	119	90	65	93	92	21–7	21–6	24–7	22–7	21–6
May	77	128	117	92	96	25–11	24–12	26–14	24–12	24–11
June	93	192	86	142	106	31–16	29–17	31–19	30–17	29–17
July	70	81	184	122	120	30–17	29–17	31–20	31–20	31–18
August	104	66	97	225	89	29–16	30–19	30–20	31–19	29–18
September	147	80	176	149	98	26–15	26–16	28–15	25–16	26–14
October	53	67	121	86	86	19–6	19–7	22–7	20–6	21–7
Total	768	833	935	1056	791					

**Table 8:** Foliar and soil N treatments used in the 2015–17 field sites.

N Treatment Designation ^1^		N Treatment Description	
		Total N Applied	N Application Times ^2^
		kg N ha^−1^	
	0	0	
Foliar N	11.2 × 1	11.2	14 d pre-veraison
	22.4 × 1	22.4	14 d pre-veraison
	11.2 × 2	22.4	14 d pre-veraison; veraison
	44.8 × 1	44.8	14 d pre-veraison
	22.4 × 2	44.8	14 d pre-veraison; veraison
	11.2 × 3	33.6	14 d pre-veraison; veraison; 5 d post veraison
	11.2 × 4	44.8	14 d pre-veraison; veraison; 5 & 10 d post-veraison
Soil N × 1		100 (2015) 200 (2016, 2017)	pre-bud break

^1^ foliar and soil applied N rates × number of applications, ^2^ veraison occurred 24 July (2015), 28 July (2016), 25 July (2017).

**Table 9:** Description of sites used in the P and K response studies (2008–2011).

Site Designation	Year Treatments Initiated	Variety	Rootstock	Year Planted	Soil Classification
P sites					
1P09	2009	Cabernet Sauvignon	3309	2000	Fine, kaolinitic, mesic Typic Kanhapludults
2P09	2009	Chamborcin	SO4	2006	Clayey, mixed, thermic Typic Hapludults
K sites					
1K09	2009	Cabernet Franc	3309	2000	Fine, kaolinitic, mesic Typic Kanhapludults
2K08	2008	Chardonnay	101–14	2004	Fine, kaolinitic, mesic Typic Kanhapludults

## Data Availability

The data presented in this study are available on request from the corresponding author.
